# Life cycle of the cold-water coral *Caryophyllia huinayensis*

**DOI:** 10.1038/s41598-023-29620-x

**Published:** 2023-02-14

**Authors:** Thomas Heran, Jürgen Laudien, Rhian G. Waller, Verena Häussermann, Günter Försterra, Humberto E. González, Claudio Richter

**Affiliations:** 1 Alfred-Wegener-Institut Helmholtz-Zentrum für Polar- und Meeresforschung, Am Alten Hafen 26, 27568 Bremerhaven, Germany; 2 University of Bremen, Bibliothekstraße 1, 28359 Bremen, Germany; 3 Fundación San Ignacio del Huinay, Casilla 462, Puerto Montt, Chile; 4 Tjärnö Marine Laboratory, University of Gothenburg, 452 96 Strömstad, Sweden; 5 Facultad de Ciencias de la naturaleza, Escuela de Ingeniería en Gestión de Expediciones y Ecoturismo, Universidad San Sebastián, Lago Panguipulli, 1390, Puerto Montt, Chile; 6 Facultad de Recursos Naturales, Escuela de Ciencias del Mar, Pontificia Universidad Católica de Valparaíso (PUCV), Avda. Brasil, 2950, Valparaíso, Chile; 7 Instituto de Ciencias Marinas y Limnológicas, Universidad Austral de Chile, Valdivia, Chile; 8 Research Center: Dynamics of High Latitude Marine Ecosystems (FONDAP-IDEAL), Valdivia, Chile

**Keywords:** Coral reefs, Behavioural ecology, Biological metamorphosis, Embryology, Population dynamics

## Abstract

Little is known about the biology of cold-water corals (CWCs), let alone the reproduction and early life stages of these important deep-sea foundation species. Through a three-year aquarium experiment, we described the reproductive mode, larval release periodicity, planktonic stage, larval histology, metamorphosis and post-larval development of the solitary scleractinian CWC *Caryophyllia (Caryophyllia) huinayensis* collected in Comau Fjord, Chilean Patagonia. We found that *C. huinayensis* is a brooder releasing 78.4 ± 65.9 (mean ± standard deviation [SD]) planula larvae throughout the year, a possible adaptation to low seasonality. Planulae had a length of 905 ± 114 µm and showed a well-developed gastrovascular system. After 8 ± 9.3 days (d), the larvae settled, underwent metamorphosis and developed the first set of tentacles after 2 ± 1.5 d. Skeletogenesis, zooplankton feeding and initiation of the fourth set of tentacles started 5 ± 2.1 d later, 21 ± 12.9 d, and 895 ± 45.9 d after settlement, respectively. Our study shows that the ontogenetic timing of *C. huinayensis* is comparable to that of some tropical corals, despite lacking zooxanthellae.

## Introduction

Cold-water corals (CWCs) are widespread across the oceans and provide important habitats for a rich associated fauna^1–3^. They exhibit a great diversity of life cycles and developmental stages. Most information on coral life cycles of CWCs is available for octocorals^4^, while only little is known about cold-water scleractinians^5^. The difficulty to observe CWCs in situ and the challenge to rear corals in aquaria systems with suitable conditions for reproduction has hampered the study of the early life cycles of CWCs.

The life cycle of scleractinian corals includes a sessile benthic phase and a mobile phase with either a benthic crawling or pelagic swimming planula^6^. The larva undergoes settlement when a permanent contact with the substrate is stablish and metamorphosis to the primary polyp^7^ where physiological and morphological transformation take place, with the formation of the basal plate initiating the calcium carbonate skeleton. Subsequently the primary polyp grows to an adult reproductive coral, thus completing the life cycle.

To date, two modes of sexual reproduction are known in adult CWC polyps: broadcast spawning and brooding. Broadcast spawners release gametes into the water column for external fertilization and development of free-swimming planula. In brooders, fertilization occurs internally and subsequent larval development takes place within the gastrovascular cavity of the polyp resulting in the release of developed planulae. Most scleractinian CWCs studied to date are broadcast spawners, including colonial hermatypic^8–11^, pseudo-colonial^12^, and solitary forms^13–16^, while brooding has been observed in three Antarctic solitary scleractinians, i.e., *Flabellum thouarsii, F. curvatum* and *F. impensum*^17^, and in the sub-Antarctic *Balanophyllia malouinensis* from the Drake Passage^18^.

Previously it was hypothesized that coral sexual reproduction is related to the life history, morphology, and habitat selection in several ways^19–21^. First, it has been noted that small polyps appear to produce small eggs and brood larvae, whereas massive or large-polyps spawn with subsequent external fertilization^22^. Second, polyps with large inter-septa spaces allow brooding, suggesting that the inter-septa spacing would be a proxy for the reproductive mode^23^. Third, brooding may have evolved in corals as an adaptation to increase recruitment success in unstable habitats, i.e., frequent physical disturbances such as storms or substrate slumping, or infrequent but catastrophic disturbances such as extreme low tides, sea warming events, and sea level changes^21^. Short larval dispersal could be beneficial for infrequent disturbances, and long larval dispersal would be beneficial for frequently disturbed habitats.

The contrasting modes of reproduction (spawning and brooding) could lead to differences in the pelagic larval duration (PLD), and consequently to different scales of larval dispersal. PLD correlates with both the duration of the pre-competency period, (i.e., the time during which larvae have no means to settle) and the competency window (i.e., when the larva is searching for a suitable substrate while being able to settle when it finds it). In broadcast spawners, the larval period is spent primarily in the water column, whereas in brooders, the planulae remain within the parent polyp (after internal fertilization) before leaving the parent and settling. If a larva takes too long to find a substrate, however it may not be able to settle and metamorphose^24^. The competency period may also depend on the feeding ability/disability of the larvae, i.e., it may be longer for planktotrophic (feeding) larvae that have a mouth and gastrovascular cavity and shorter for lecithotrophic (non-feeding) larvae. The larval period is the most vulnerable part of the life cycle due to the presence of pelagic predators as well as environmental threats. Laboratory studies on *Desmophyllum pertusum* found a pre-competency period averaging 3–5 weeks^25^. While the PLD has not yet been determined, it has been assumed to range from 99 d to one year^5^. The long PLD is considered to be the result of the planktotrophy of *D. pertusum* larvae^5^. Instead, in the facultatively photosymbiotic *O. varicosa*, the pre-competency period and PLD are shorter, lasting between 1 and 2 weeks and between 21 and 27 d^9^, respectively. Although the feeding behaviour of *O. varicosa* larvae is still undetermined, laboratory observations suggest a lecithotrophic larva^26^.

Larval settlement in scleractinians is triggered by endo- and/or exogenous cues^27^. Exogenous chemical cues from algae^27^ and microbial biofilms^28^ are detected by specialized cilia or flagella at the aboral epidermis^29^, i.e., the apical organ or apical tuft. After apical contact with the substrate, nematocysts discharge and mucus of the epidermis forms an adhesive plug^30^. This is followed by contraction of the oral-aboral plane, resulting in a flattened disc subdivided by radial mesenteries. In tropical corals, this process has been well studied^31,32^; however, in CWCs, there are few descriptions of the early life cycle^5^.

*Caryophyllia (Caryophyllia) huinayensis* Cairns et al.^33^ is a small (20 mm high, 10 mm ø)^33^ solitary azooxanthellate scleractinian recorded in Chilean waters between 36° and 48.5° S^33^. In Comau Fjord, Chile, it is found between 11 and at least 265 m depth^34,35^ (revealed from a remotely operative vehicle (ROV) until half way down in Comau Fjord), while on the continental slope it occurs between 740 and 870 m water depth^36^. Despite being an important representative of the benthic biodiversity of the Patagonian fjords, reaching densities of up to 2211 ± 180 ind. m^−2^ at 25 m water depth on steep and overhanging walls^34^, its reproductive biology and life cycle are so far unknown.

The objectives of this study were to describe (i) reproductive mode and periodicity, (ii) larval behaviour and features, and (iii) metamorphosis and post-larval development of the scleractinian CWC *C. huinayensis*. For this purpose, reproductive polyps were reared in a holding tank over three years, where released larvae were collected periodically to determine the production rate. Brooded larvae were characterized through histological cuts of the polyps. Collected larvae were introduced into flow-through glass cubes where the early life cycle was monitored from the planktonic phase through settlement, metamorphosis and growth to a young polyp.

## Results

### Reproductive mode and periodicity

**Figure 1:**
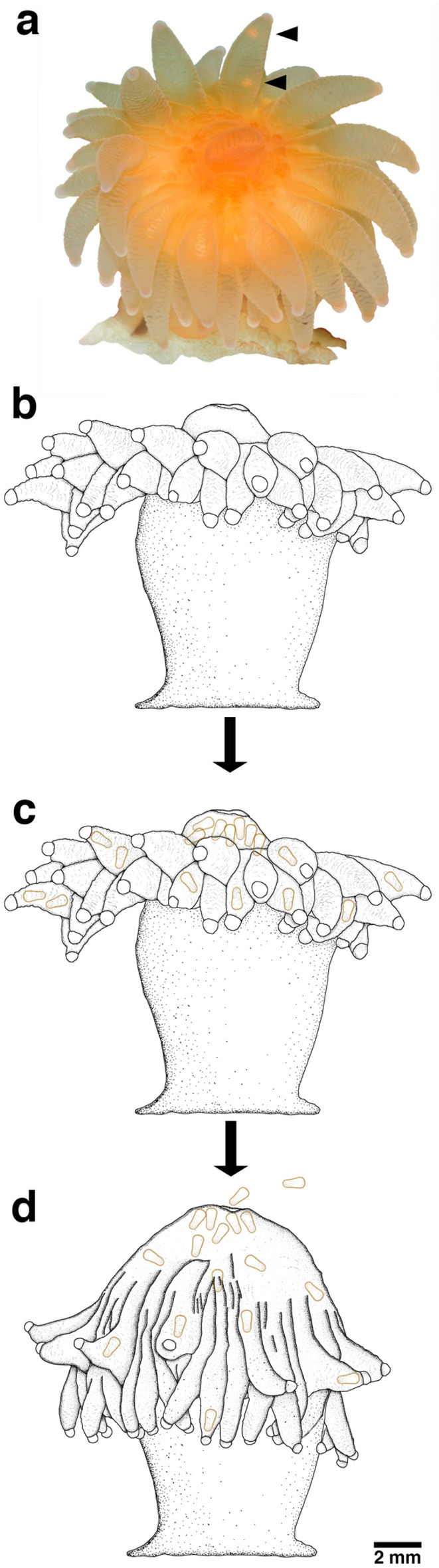
Temporal sequence of larval release of *Caryophyllia huinayensis.* (**a**) reproductive polyp with two larvae (black arrowheads) in a tentacle. Schematic drawing of (**b**) a polyp with larvae not visible or absent, (**c**) polyp with larvae in the gastrovascular cavity and tentacles, and (**d**) forming the dome shape before releasing larvae (in orange) through the mouth.

For this study, seven reproductive polyps of *C. huinayensis* (Fig. 1a) were extensively monitored for 3.1 years, recording the number of released larvae twice per day. Although no sperm release was observed throughout the entire study, larvae could be observed across the translucent oral tissue of the polyps swimming in the coelenteron beneath the oral disk and tentacles. A total of 1647 planulae were released from the polyps into the surrounding water, from which 19 larvae that settled and metamorphosised into young polyps were monitored.

It is likely that only part of the larvae in the coelenteron were counted as planulae swimming at a distance from the tissue surface may have remained undetected. Periods with no visible larvae (Fig. 1b) were generally followed by the sudden appearance of 2–3 larvae swimming from one tentacle to another. Subsequently, the number of larvae increased in the gastrovascular cavity below the oral disc and in the tentacles, reaching up to 17 planulae per polyp (Fig. 1c). As brooding progressed, polyps changed shape: the oral disc bulged outward until assuming a dome shape lasting 1–2 d which was ended by a ~ 10-s contraction causing the gastrovascular fluid containing the larvae to exit through the mouth (Fig. 1d). Occasionally, we observed larvae released at time when corals were feeding on krill.

**Figure 2:**
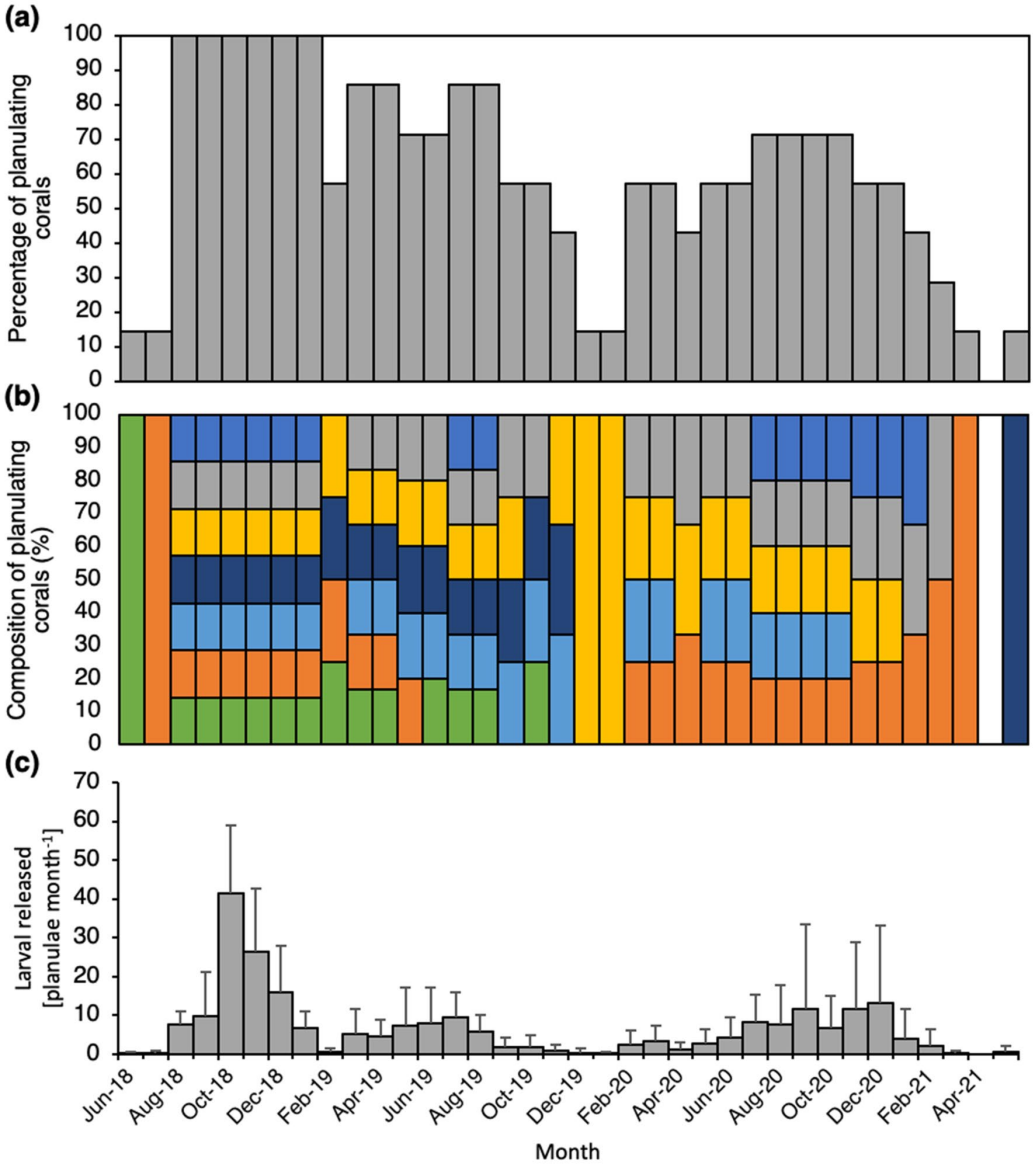
Seasonal and interannual variability of the planulation activity of *Caryophyllia huinayensis*. (**a**) Percentage of planulating corals, (**b**) composition of planulating corals, with color-coded bars representing each of the seven parental polyps included in the study. (**c**) Larval release rate (mean ± SD) per month among all parental polyps. Note: No larvae were released in April 2021.

*Caryophyllia huinayensis* individuals released 78.4 ± 65.9 (mean ± SD) larvae per year, with the exception of April 2021 (Fig. 2a–c). Synchronous spawning of all 7 polyps was observed from August 2018 to January 2019 (Fig. 2b). Thereafter, the reproductive activity showed a bi-modal distribution: where 70–80% of the polyps released planulae in austral fall and winter (March–August 2019), and austral winter and spring (June–October 2020), in contrast to austral summer (December 2019–January 2020) and spring (March–May 2021), where only 0–13% of the polyps released larvae (Fig. 2a).

**Figure 3:**
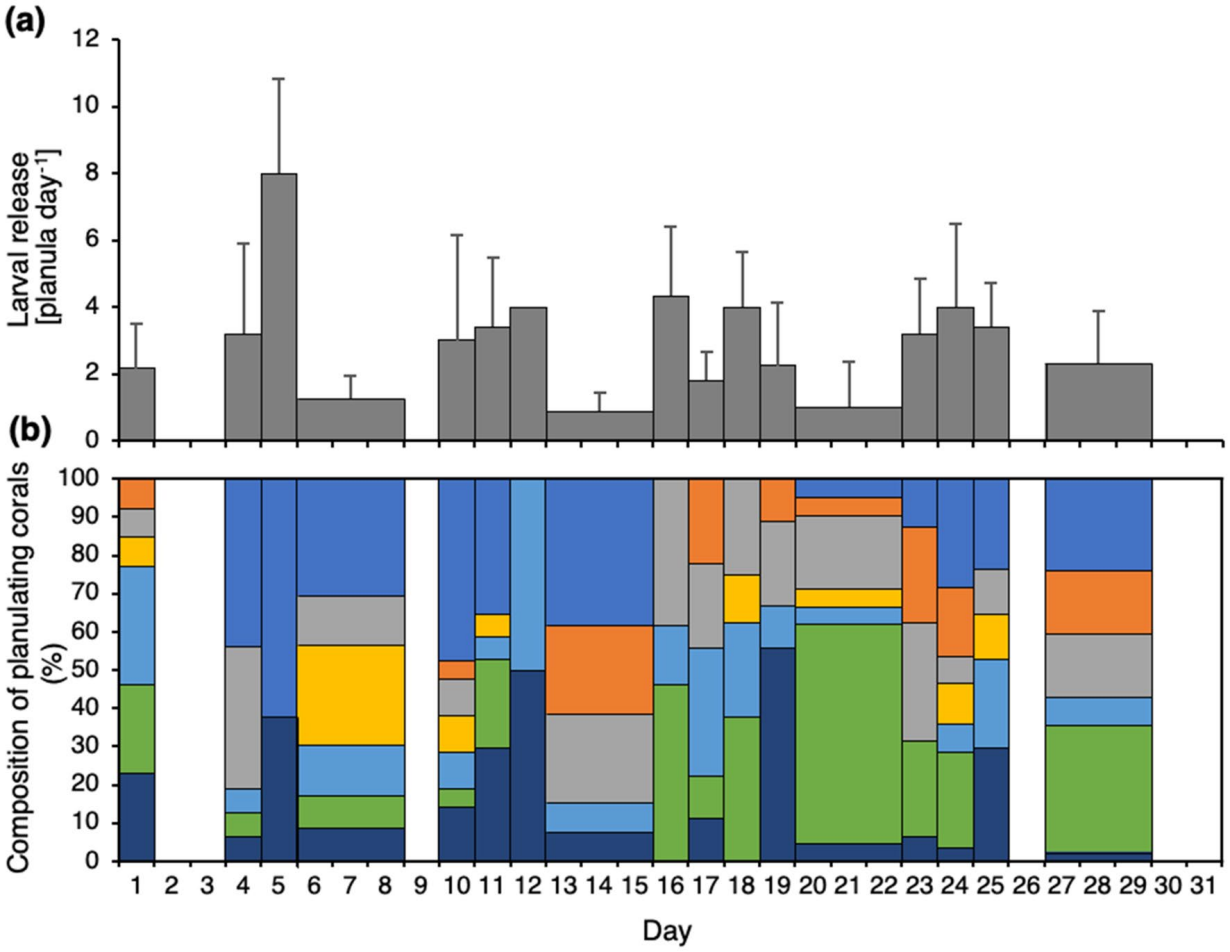
Daily larval release among the seven *Caryophyllia huinayensis* polyps during the most active month (October 2018). (**a**) Planula per coral and daily release rate (mean ± SD), and (**b**) composition of planulating corals, with the color-coded bars representing each of the seven parental polyps included in the study. Note: Broad bars indicate values averaged over the weekend, as larvae were monitored from Monday to Friday; no larvae were released on 2nd, 3rd, 9th, 26th, 30th, 31st of October 2018.

The proportion of corals engaged in the production and release of larvae was reflected in larval numbers (Fig. 2c). We observed three peaks of released larvae: one in austral spring (October 2018) with > 40 larvae polyp^−1^ month^−1^, and two broader ones in austral winter (June–July 2019) and spring (September–December 2020), both with < 15 larvae polyp^−1^ month^−1^ (Fig. 2c). The maximum number of released larvae was observed during October 2018, with a total of 43 larvae polyp^−1^ month^−1^ (Fig. 3b), with a maximum of 14 released larvae by one polyp in one day (Fig. 3a). In November 2020, a single polyp released 21 larvae in one day. Monthly larval release differed temporally, with some polyps pausing planulation for 6.1 ± 5.9 consecutive months, with a maximum of 17 months. Compared to polyps that continuously planulated for 11.7 ± 3.4 months, with a maximum of 16 months (Fig. 2b).

### Larval features and behaviour

Larvae were 905 ± 114 µm in length, 371 ± 48 µm in width (n = 12), with a body shape that varied between cylindrical, elongated and curved within minutes when swimming. A teardrop-shaped form was acquired when the apical side was in contact with the substrate.

**Figure 4:**
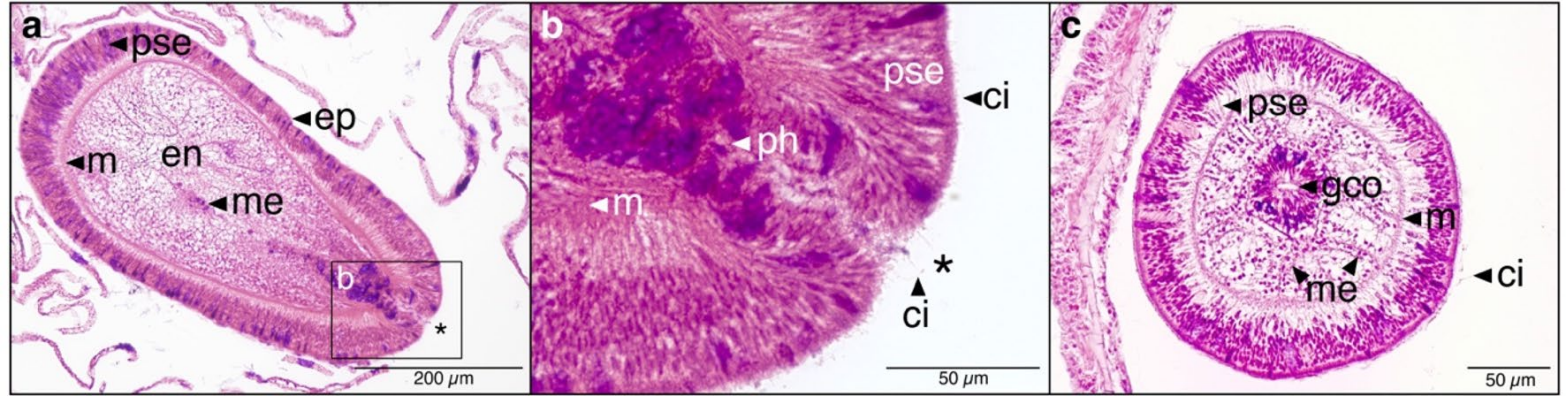
Haematoxylin-eosin-stained longitudinal (**a**, **b**) and transverse (**c**) histological sections of two larvae inside a polyp of *Caryophyllia huinayensis*. (**a**) Planula showing the epidermis (ep), the pseudo-stratified epidermis (pse), a thin layer of mesoglea (m), and the endodermis (en). Note that the mesenterial filaments (me) are attached to the pharynx (ph), which in turn is connected to the blastopore/oral pore (*), shown in detain in (**b**). (**b**) Detail from (**a**) showing a ciliated (ci) oral pore connected to the pharynx. (**c**) The gastrovascular cavity opening (gco) and the uniform ciliated epidermis are visible.

Histologically, the larvae featured the typical bilayered tissue with a uniformly ciliated (ci) epidermis (ep), a thin layer of mesoglea (m), and an endodermis (en) with mesenterial filaments (me) which attach to the pharynx (ph), i.e., the muscular throat region (Fig. 4a,b). Like other cnidarian larvae, the aboral end likely holds the sensory organ (or the apical organ), being a pseudo-stratified epidermis (pse) with sensory cells that detect the exogenous chemical cues for settlement (Fig. 4a). On a total of 25 larvae, the pharynx at the oral end connected the blastopore/oral pore (*) (Fig. 4b) with the gastrovascular cavity opening (gco) (Fig. 4c).

**Figure 5:**
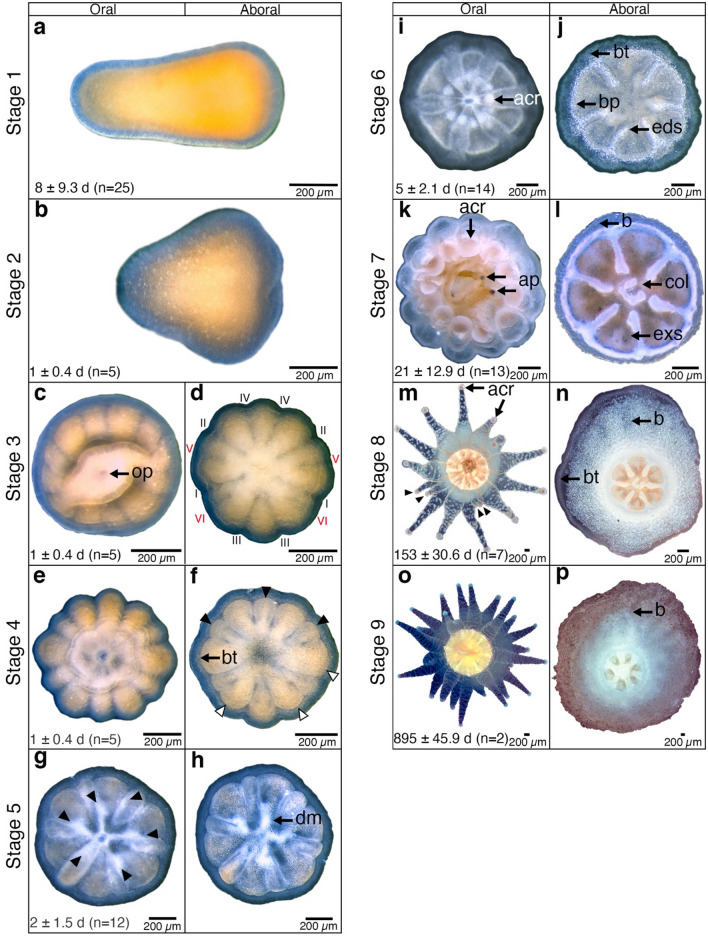
*Caryophyllia huinayensis* from larva to juvenile polyp (compiled from three recruits) observed under a stereomicroscope. Columns indicate the perspective (left oral/right aboral), and rows show the number of days (mean ± SD) from attachment to reach the respective stage. For description of Stages 1–9, see main text. acr: acrosphere, ap: *A. persimilis*-nauplii, b: base, bp: basal plate, bt: bilayered tissue, col: columella, dm: directive mesenteries, eds: endosepta, exs: exosepta, op: oral pore. In all photographs, the background was replaced with white for better visualization (free-form selection).

During the first two days after release, most larvae swam near the water surface, but for the remainder of the larval period they swam mainly near the bottom, where no benthic crawling was observed. Swimming occurred in the apical direction with a superimposed counterclockwise rotation, so that the planula moved forward in a corkscrew-like manner with a swimming velocity of 1.18 ± 0.4 mm s^−1^ (n = 12). Attachment was sometimes followed by detachment until successful settlement and subsequent metamorphosis. If temporary attachment occurred, the planula detached after a maximum of 24 h and started its searching behaviour again. The planktonic stage lasted 8 ± 9.3 d (Stage 1, Fig. 5a) when settlement surfaces were preconditioned. However, larvae exposed to non-preconditioned surfaces had a longer PLD, 26.7 ± 2.1 d (n = 4). Three of four of these eventually settled, and one died after 29 d without settling. Larvae attached on coverslips near microscale topographic features, such as silicon ridges or cracks in the coverslip glass. In all cases, settlement was limited to the overhanging surfaces, although larvae released into the holding tank settled on both the floor and walls.

### Metamorphosis and post larval development

**Figure 6:**
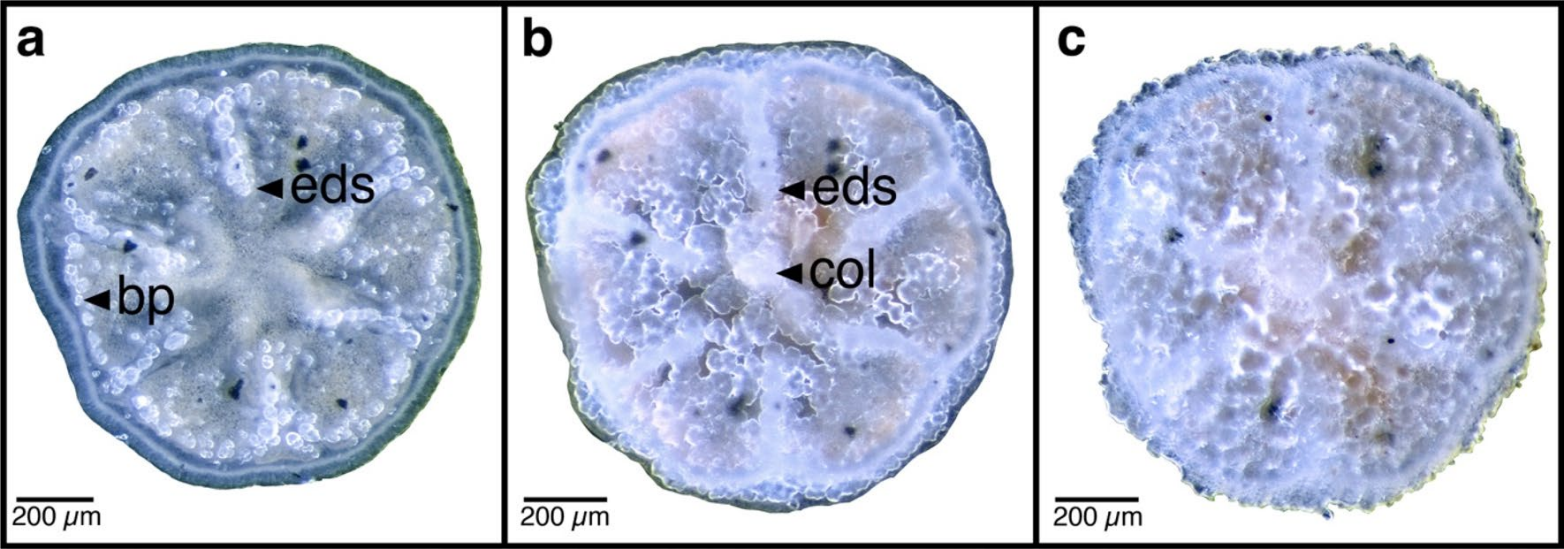
Aboral side of a settled *Caryophyllia huinayensis*; (**a**) 6 d, (**b**) 32 d and (**c**) 84 d after settlement. Skeletogenesis begins with the formation of a basal plate ring and the endosepta (**a**), and light scattering of the growing crystals result in an increasingly opaque appearance (**b**). Finally, the first set of endosepta is barely visible and the first set of exosepta is no longer visible, nor is the columella (**c**). All images were taken from the same coral and the background was replaced with white for better visualization (free-form selection). bp: basal plate, col: columella, eds: endosepta.

The time of each metamorphic stage was calculated based on the number of days since larval attachment. After attachment to the substrate, larvae flattened (Stage 2, Fig. 5b), whereupon the blastopore/oral pore (op) became visible as an oval opening (Stage 3, Fig. 5c). Stage 3 revealed the six body divisions (mesenteries), characteristic of the sixfold symmetry in the body plan of Hexacorallia. Six pairs of primary proto-mesenteries, the protocnemes (sensu Duerden^37^), divided the polyp radially in the longitudinal plane, extending from the aboral base to the oral pore and pharynx (Fig. 5d; I–VI). Of these protocnemes, four were incomplete, as were the corresponding divisions (Fig. 5d; V, VI in red). In Stage 4, the divisions of the protocnemes were more developed and alternately formed three pairs of entocoel (Fig. 5f; black arrowheads) and exocoel chamber (Fig. 5f; white arrowheads). At this stage, larvae settled and the ectoderm and the endoderm formed the bilayered tissue (bt) (Fig. 5f). In Stage 5 (2 ± 1.5 d), the first set of tentacles, called endotentacles (Fig. 5g; black arrowheads), appeared on the wall of the entocoel chamber as white nodular tissue formed around the oral pore. On the aboral side, the directional mesenteries (dm) were observed as four whitish branches connected to the endotentacles, which could contract (Fig. 5h). In Stage 6 (5 ± 2.1 d), the second set of tentacles, the exotentacles occurred on the wall of the exocoel chamber (Fig. 5i). The 12 tentacles (endo- and exotentacles) developed an acrosphere (acr), i.e., globular tip rich in cnidae (stinging organelles) (Fig. 5i,k,m). At this stage, the first skeletal elements of the basal plate (bp) formed along the periphery of the polyp and extended radially inwards along the six primary septa, the endosepta (eds) delimiting the entocoel chamber (Fig. 5j). At Stage 7 (21 ± 12.9 d), acrospheres were well developed and the tentacles could be extended to catch food (*A. persimilis*-nauplii (ap)) (Fig. 5k). The basal plate and primary septa thickened, resulting in a lateral fusion of the two. The second set of exosepta (exs) appeared (Fig. 5l), and the U-shaped columella (col) protruded from the centre of the basal plate (Fig. 5l). At this stage, in three of the eight recruits, the crystals grew in different directions, which created an opaque layer, impairing the observation of the columella and the primary and secondary septa (Fig. 6). Also, at this stage the bilayered tissue (bt), the endoderm and ectoderm layers, left the base (b) uncovered (Fig. 5l), in contrast to the former stage (Fig. 5j). In Stage 8 (153 ± 30.6 d), the first and the second set of tentacles had visible nematocyst in the form of white packages surrounding the tentacles, and the third set of tentacles appeared between the exotentacles and entotentacles (Fig. 5m, black arrowheads). The bilayered tissue again covered the base (Fig. 5n). In Stage 9 (895 ± 45.9 d), the fourth set of tentacles developed, leading to a total of 24 tentacles (Fig. 5o). The basal plate thickened, the entocoelic septa and columella merged (Fig. 5p), and the bilayered tissue retracted from the base again (Fig. 5p). No post-settlement mortality was observed during the 3.1-year study.

## Discussion

The solitary cold-water scleractinian *C. huinayensis* is described here as a brooder. Although reproduction in scleractinian CWCs is still poorly known, no other temperate species has yet been described to brood larvae. The solitary temperate CWC *D. dianthus*^12^, as well as the temperate colonial CWC *D. pertusum*^8,10,38^*, M. oculata*^8,10^ and *O. varicosa*^9^ reproduce by broadcast spawning. Brooding has only been reported in subpolar and polar solitary CWCs from the Southern Ocean^17,18^.

Although quantitative data on the number of larvae released in the four Southern Ocean brooders are lacking, a potential number of larvae released per polyp can be inferred from their maximum fecundity (Table 1). *C. huinayensis* appears to be in the lower range of larvae production (6.5 ± 11.4 month^-1^ larvae), when compared its larval size (750–1080* µm* length) with *Balanophyllia malouinensis* larvae (> 600 µm, Table 1).

**Table 1:** Larval features of scleractinian CWC species.

Species	Larval length (µm) min–max	Swimming velocity (mm s^-1^) min–max	Maximum fecundity Oocyte per polyp (mean ± SD)	Reproductive mode	Formation	Geographic distribution*	References
*Balanophyllia elegans*	3000–5000	0.004–0.006	n.d	Brooder	Solitary	Northeast Pacific	Fadlallah and Pearse^23^, Gerrodette^39^
*Balanophyllia malouinensis*	> 600	n.d	241 ± 181	Brooder	Solitary	Sub-Antarctic	Pendleton et al.^18^
*Caryophyllia huinayensis*	750–1080	0.35–1.18	n.d.**	Brooder	Solitary	Chile	This study
*Caryophyllia smithii*	800–1000	ca. 0.017–0.05	n.d	Broadcast spawner	Solitary	Atlantic Ocean, Mediterranean sea, Indo-West Pacific	Tranter et al.^40^
*Desmophyllum pertusum*	120–270	0.29–0.86	3146 ± 1688	Broadcast spawner	Colonial	Cosmopolitan	Larsson et al.^41^, Waller and Tyler^10^
*Flabellum angulare*	2000–3000	n.d	550	Broadcast spawner	Solitary	Eastern Central Pacific, Northeast Atlantic	Mercier et al.^15^
*Flabellum curvatum*	n.d	n.d	1618 ± 1071	Brooder	Solitary	Antarctic, Southwest Atlantic, Southeast Pacific	Waller et al.^17^
*Flabellum impensum*	n.d	n.d	1270 ± 884	Brooder	Solitary	Antarctic	Waller et al.^17^
*Flabellum thouarsii*	n.d	n.d	2412 ± 1554	Brooder	Solitary	Antarctic, Southwest Atlantic	Waller et al.^17^
*Oculina varicosa*	217	1.6	1000–4800 (cm^-2^ colony)	Broadcast spawner	Colonial	Western Central Atlantic	Brooke and Young^9^, Brooke and Young^26^

n.d. = no data.

*According to Palomares and Pauly^42^.

**Publication in preparation.

Thorson’s rule^43,44^ states that organisms at higher latitudes tend to produce larger and fewer offspring and are frequently brooders. However, the brooding *C. huinayensis* appears to defy this rule, as it occurs at mid-latitudes (36° and 48.5° S^33,45^). Though the phylogeography of *C. huinayensis* is not yet clear, six other solitary species of the genus *Caryophyllia* are endemic to Antarctica^46^, suggesting that the genus may have originated in the Southern Ocean, with the mid-latitude distribution of *C. huinayensis* making the downstream end dispersal via the cold Humboldt Current branching off the Southern Ocean. In our case, Thorson’s rule does not seem to be a good predictor of the macroevolutionary patterns and reproductive mode in *Caryophyllia*.

A better explanation can be inferred from Kerr et al.^47^. Their phylogenetic study on scleractinians revealed that the change from spawning to brooding (or vice versa) is based on the sexuality of the corals (i.e., gonochoric or hermaphroditic) and not on latitudinal distribution. The main pathway is from gonochoric spawners to gonochoric brooders, then to hermaphroditic brooders, and finally hermaphroditic spawning, which is the dominant reproductive mode in shallow-water corals.

The results of our study indicate that *C. huinayensis* reproduces throughout the year, albeit with large temporal variations in the number of larvae released. However, the fluctuations were not seasonal. This may be due to the fact that the aquarium for this experiment lacked external timing signals (zeitgebers) usually found in the field, i.e., there were no fluctuations e.g., in water temperature, food frequency, food quality, or salinity, which might otherwise have synchronized the corals’ internal clock. Although, it is not yet known if the local *C. huinayensis* population exhibits seasonality in their larval release, the lack of larvae in April 2021 could also be due to poor internal fertilisation success based on the quality and/or quantity of sperm released (which was never observed).

If there is no seasonal release of larvae from the natural coral population, this may indicate that rapid recolonisation is possible throughout the year following a disturbance. Substrate alterations are usually observed in the Patagonian fjord region, where strong physical disturbances such as landslides occur^48^, due to precipitation and earthquakes^49^. Also, hypoxia events (< 2 ml l^-1^) caused by pulses and subsequent degradation of terrestrial and marine organic matter (e.g., phytoplankton blooms)^50,51^ are common in the fjords^51^, negatively impacting the benthic life, but promoting new available space for settlement. These events may be exacerbated in time and space by salmon farming activities, as bacterial respiration during the degrading of uneaten food pellets or salmon faeces^52^, decreasing the oxygen concentration and thus reduces the likelihood that recolonisation can take place at a natural rate after hypoxic events.

On the one hand, brooding in *C. huinayensis* with subsequent release of well-developed larvae throughout the year has the advantage of rapid settlement, reducing the likelihood of drift to unfavourable locations and die-off^21^. On the other hand, a high dispersal potential has a positive effect, as larvae can spread to new habitats with better conditions. Therefore, stable and suitable environmental conditions at a site, with occasional disturbances that expose substrates are favorable for brooder with short larval dispersal, as has been shown for *C. huinayensis*. However, if environmental conditions vary, longer-dispersed larvae may have an advantage. Different reproductive approaches were observed in two brooding tropical *Agarcia*-species from a reef off Curaçao, where *A. humilis*—which occurs in shallow waters subject to physical and biological disturbance—has many small larvae throughout the year. In contrast, *A. agaricites* that thrives in deeper and less disturbed areas, has few but large larvae, which are released only in spring/summer^20^. While the low fecundity and narrow time window for reproduction limit the species’ capacity to colonise new available substrate, the disadvantage is compensated by the larger size and, hence, long-lasting energy reserves of the larvae, promoting higher dispersal.

As access to natural CWC populations is difficult and the release of offspring can rarely be observed, it is difficult to establish a link between reproduction and environmental cues. However, Maier et al.^53^ conducted a transplantation experiment with *D. pertusum* at Nakken reef, Norway, determining that gametogenesis is supported by lipids obtained during spring, profiting from increased phyto- and zooplankton abundance at this time. The coral then rebuilt the tissue reserves in autumn, probably related to the spawning season (late January to early March)^38^.

Spawning during the winter season was observed for the actinia *Corynactis* sp.^54^ and suggested for *D. dianthus*^12^ and the octocoral *Primnoella chilensis*^55^, all from Comau Fjord. This period coincides with the seasonal temperature minimum^56^ at the sampling depth of *C. huinayensis*. It is thus conceivable that the change in water temperature initiates gametogenesis, while increasing food availability in early spring fosters the development and release of brooded planulae. The peak of released larvae of in vitro-reared *C. huinayensis* (June–December 2018, Fig. 2c) matches the reproductive season of *D. dianthus* and *P. chilensis*. Although water temperature and food supply were kept constant in our laboratory study, an endogenous clock can be suspected as a pacemaker for reproduction that the adult polyps collected in the field “remembered” but run out of phase due to the lack of an external natural stimulus, which may explain the observed variability in planulation (Fig. 2c).

The swimming behaviour of *C. huinayensis* larvae in the first days was similar to that of *O. varicosa*^26^ from an in vitro experiment, in which the larvae spent two to four days at the water surface and three weeks in the lower water column before metamorphosing*.* Likewise, *D. pertusum* larvae remained at the water surface for the first two weeks before bottom-probing 3–5 weeks after fertilization^41^. Translating the in vitro observations of *C. huinayensis* larvae to the field, this might indicate that the two days of swimming upwards and being pelagic do not provide much potential for dispersal. However, the strong tidal currents of the photic zone^49,57^ could significantly increase their dispersal. It should be noted, however, that in Comau Fjord a strong halocline at around 10–15 m depth probably prevents the larvae from swimming up to the brackish surface layer (7–31 salinity)^33,58^. This however needs to be verified, as larvae of *D. pertusum* are able to survive at a salinity of 25^5^.

One of the forces governing connectivity between coral populations is thought to be related to the planktonic stage, though not solely based on larvae locomotion. At 1.18 ± 0.4 mm s^-1^, the swimming velocity of *C. huinayensis* larvae is within the range of other CWCs (Table 1). The determined swimming speed of *C. huinayensis* is, however, 1–2 orders of magnitude lower than the tidal current velocities recorded at the vicinity of coral populations in Comau Fjord (5 cm s^-1^, maximum 15 cm s^-1^)^59^. Hence, horizontal dispersal is likely dominated by drift, as in other planktonic larvae, and the range is largely determined by the PLD ended by settlement. Former studies found that larvae released from brooders can settle within hours or a few days^60^, as the larvae are released at an advanced stage of development and may therefore already be competent to settle. In contrast, the entire larval stage of broadcast spawners occurs in the water column. These contrasting reproductive modes may result in different patterns of larval dispersal. For the broadcast spawners *O. varicosa*, the PLD is 42 d^9^, whereas for *D. pertusum* (where PLD was not yet described) a PLD of eight weeks made the dispersal modelling done by Fox et al.^61^ match the genetic population structure better than other models, indicating a PLD of eight weeks plausible. Thus, the potential of local settlement of these two species can be considered low due to the relatively long planktonic phase, in contrast to the 8 ± 9.3 d for *C. huinayensis*. However, studies on both sides of the Atlantic have shown a constrained genetic connectivity in *D. pertusum* between the two sides. In an investigation near Oslofjord, Norway, Dahl et al.^62^ showed that restricted connectivity also applies to populations of *D. pertusum* at a local scale. Likewise, Morrison et al.^63^ observed significant genetic differences between populations of the Gulf of Mexico and populations of the West and East Atlantic Ocean, though there is high connectivity within the regions. Populations on the European continental margin and in isolated fjords showed moderate levels of gene flow^64^. A microsatellite study on populations from Comau Fjord found no vertical or horizontal genetic differences and concluded that the local population of *D. dianthus* is panmictic^65,66^. Based on the strong tidal current in Comau Fjord, which may transport larvae both vertically and horizontally, and the relatively short planktonic phase of *C. huinayensis* (8 ± 9.3 d), the population of *C. huinayensis* may also be panmictic. However, genetic analyses would have to be carried out to elucidate the population structure of the species to be able to assess the degree of connectivity among the populations. Overall, the levels of connectivity between coral populations appears to be the interaction between large-scale currents, local environmental conditions and the species-specific reproductive modes.

When the settlement surfaces in the holding tank were allowed to grow biofilms two weeks before the start of the settlement trial, 48% of the larvae settled within one to three days. In contrast, if the surfaces were not pre-conditioned, the PLD extended up to 28 d with an average of 26.7 ± 2.1 d (n = 4). Although the number of larvae able to extend the PLD in this study is too low to draw any robust conclusions, this could nevertheless indicate that the biofilm plays a role as a cue for settlement in *C. huinayensis* larvae. This observation is supported by Webster et al.^28^, indicating that biofilms may outstrip the importance of other settlement cues (e.g., coralline algae) in shallow-water corals from the Great Barrier Reef.

Our results may imply that *C. huinayensis* larvae are able to extend their PLD when, for example, an appropriate substrate is unavailable. Consequently, PLD extension requires either energy stores (lecithotrophic larvae) or feeding (planktotrophic larvae), as shown for *D. pertusum* larvae. Here, larvae can feed on picoplankton by ciliary feeding or scavenging on mucus strings^5^. Although feeding of *C. huinayensis* larvae was not determined in this study, two lines of evidence suggest planktotrophy: the PLD may be long (26.7 ± 2.1 d), suggesting storage-independent development. More importantly, the histological cuts of 25 larvae showed a well-developed oral pore which connects to the pharynx through the gastrovascular cavity opening (Fig. 4), which may indicate a developed gastrovascular system.

Field studies on *C. huinayensis* from Comau Fjord showed high coral abundance (2211 ± 180 ind. M^2^) at 25 m water depth on a substrate inclination between 60° and 80°, but no individuals on horizontal substrate surfaces^34^. This pattern was attributed to sedimentation, where the horizontal seafloor is smothered by inorganic terrigenous particles derived from river run-off^33,58^ and/or by organic matter from intense salmon farming in Comau Fjord^67^ (such as fish faeces and uneaten food pellets passing through the salmon cages)^68,69^. Inclined surfaces, by contrast, are too steep for the sediment to accumulate. Based on the fact that the observations in this study were carried out in sediment-free artificial sea water, this demonstrates that settlement on horizontal surfaces can occur, supporting the assumption that the distribution pattern of *C. huinayensis* in the field is likely conditioned by sedimentation.

As opposed to most larvae of shallow-water scleractinians, the larvae of *C. huinayensis* lack photosynthetic endosymbionts, so that they depend entirely on heterotrophy and/or their energy reserves. Anyhow, we observed a similar structuring and timing of tissue and skeletal development in *C. huinayensis* as in the tropical corals *Galaxea fascicularis* and *Acropora brueggemanni* (re-described as *Isopora brueggemanni*)^31,32^. Within the first three days after settlement, all three coral species developed four pairs of mesenteries, which reached the stomodaeum, while two pairs did not (Fig. 5d). Moreover, the first endotentacles (2 ± 1.5 d, Fig. 5g) and the second exotentacles (4 ± 2.1 d, Fig. 5i) appeared at the same time and place in all three species. Similarly, the first crystals and crystal structures of the basal plate ring and the endosepta were precipitated (4 ± 2.1 d, Fig. 5i). These similarities suggest that early development in *C. huinayensis* is highly conserved, whether the energy costs invested during metamorphosis and juvenile growth are covered by internal energy reserves or heterotrophic feeding, or a combination of both. Although no studies have addressed the relationship between internal lipid and protein reserves with growth rate during the early growth stages in CWCs, it has been observed in the facultatively mixotrophic *O. varicosa*, where deep azooxanthellate colonies, subsisting on both energy reserves and exogenous food, grew at a higher rate than zooxanthellate shallow-water colonies^70^. This indicates that under certain conditions a heterotrophic diet may be energetically better that a mixotrophic diet.

This study was the first to describe the metamorphosis of a temperate scleractinian cold-water coral, using *C. huinayensis* as the model species. Our results show a similar ontogenetic timing from planula to juvenile polyp in *C. huinayensis* as described for tropical corals, suggesting a highly conserved evolutionary mechanism in spite of large environmental (e.g., temperature) differences.

The CWC *C. huinayensis* showed a peak in larval release in vitro*,* that coincided with the low temperatures and increased food availability at its site of origin, Comau Fjord, in austral winter. As the released larvae had a developed gastrovascular system, this could indicate a planktotrophic or mixed mode larvae, that may benefit from the increase of phyto- and zooplankton in austral spring. On the other hand, the short PLD may have the advantage of feeding immediately after settlement by offsetting the high energy costs during metamorphosis, allowing a rapid onset of skeletogenesis.

Climate change influences seasonal environmental variability by enhancing water stratification, reducing the depth of the mixed layer and water circulation, which combined, reduces the productivity of phytoplankton and consequently of the zooplankton^71^. Overall, it can alter the phenology of organisms, i.e., timing of their seasonal activities^72^. Our study showed that *C. huinayensis* displays a moderate phenology, which could be a consequence of the lack of environmental variability in the aquarium, indicating the ability of *C. huinayensis* to acclimatize to new environmental conditions. However, if the phenology of this coral is pronounced in the field, the expected changes in food availability would not match the observations in the aquarium. On the contrary, lower energy uptake could therefore affect reproductive capacities, larval survival, metamorphosis and growth, impairing the connectivity and thus population stability of this species.

## Materials and methods

### Collection of specimens

**Figure 7:**
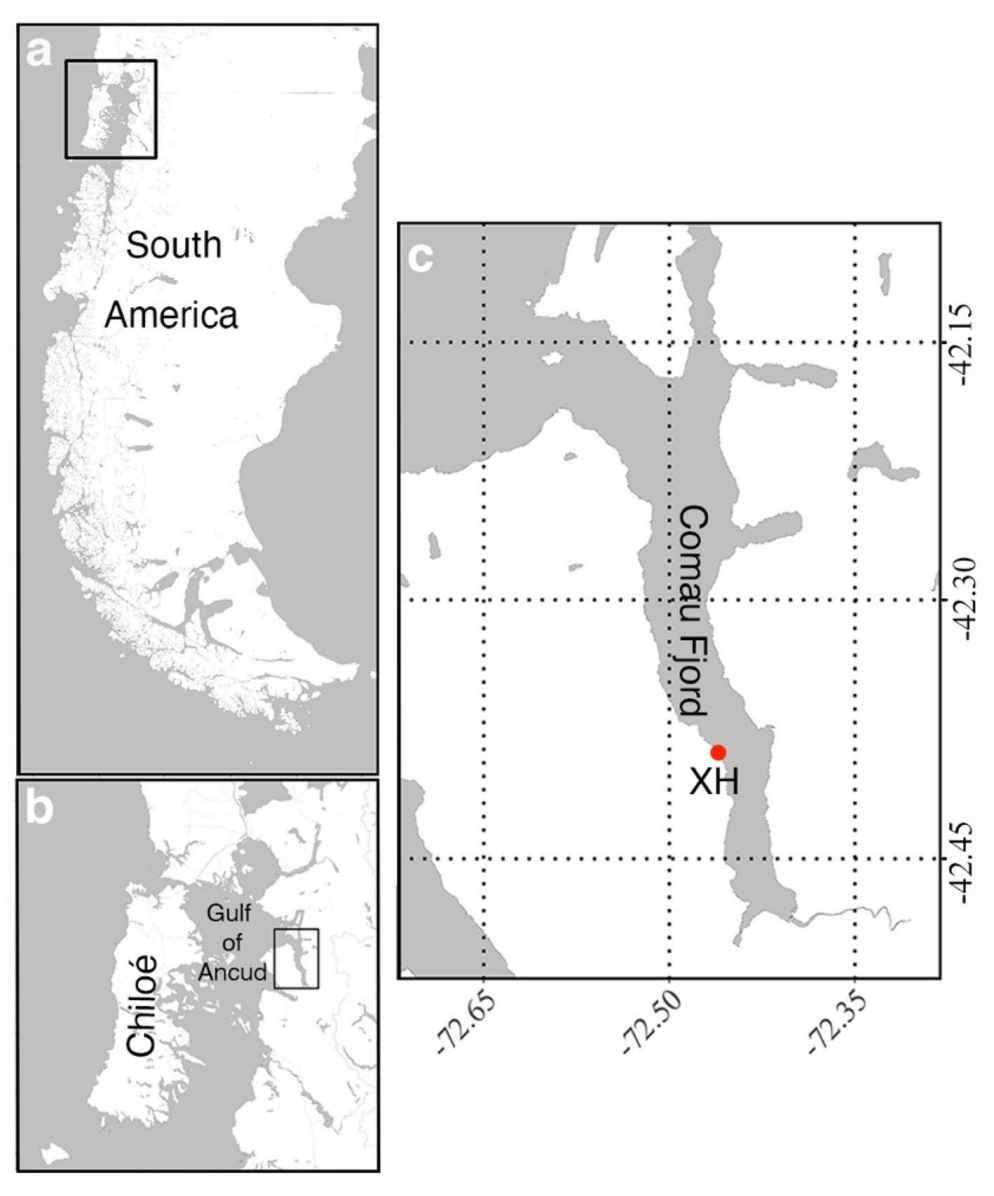
*Caryophyllia huinayensis* collection site. (**a**) Overview of the Patagonia. Black rectangle denotes the area in **b.** (**b**) Chiloé island and Gulf of Ancud with Comau Fjord (black rectangle). (**c**) Comau Fjord with the collection site X-Huinay (XH), where corals were sampled (red dot).

A total of 169 adult individuals of *C. huinayensis* with a length of 8.3 ± 2.0 mm and a diameter of 7.9 ± 1.4 mm were collected in 2014, 2015, and 2019 by SCUBA diving between 20 and 30 m water depth in X-Huinay (42° 23.213’ S; 72° 27.772’ W), Comau Fjord, Chile (Fig. 7). For a detailed description of the study area see Beck et al.^73^, Rossbach et al.^74^ and Sobarzo^57^. Of these corals, 60 were preserved in 4% buffered (sodium borate) formalin seawater solution for histological analysis. The remaining corals were transported alive in plastic bags filled with one-third with seawater and two-thirds with pure oxygen to the Alfred Wegener Institute in Bremerhaven, Germany.

### Maintenance specimens

**Figure 8:**
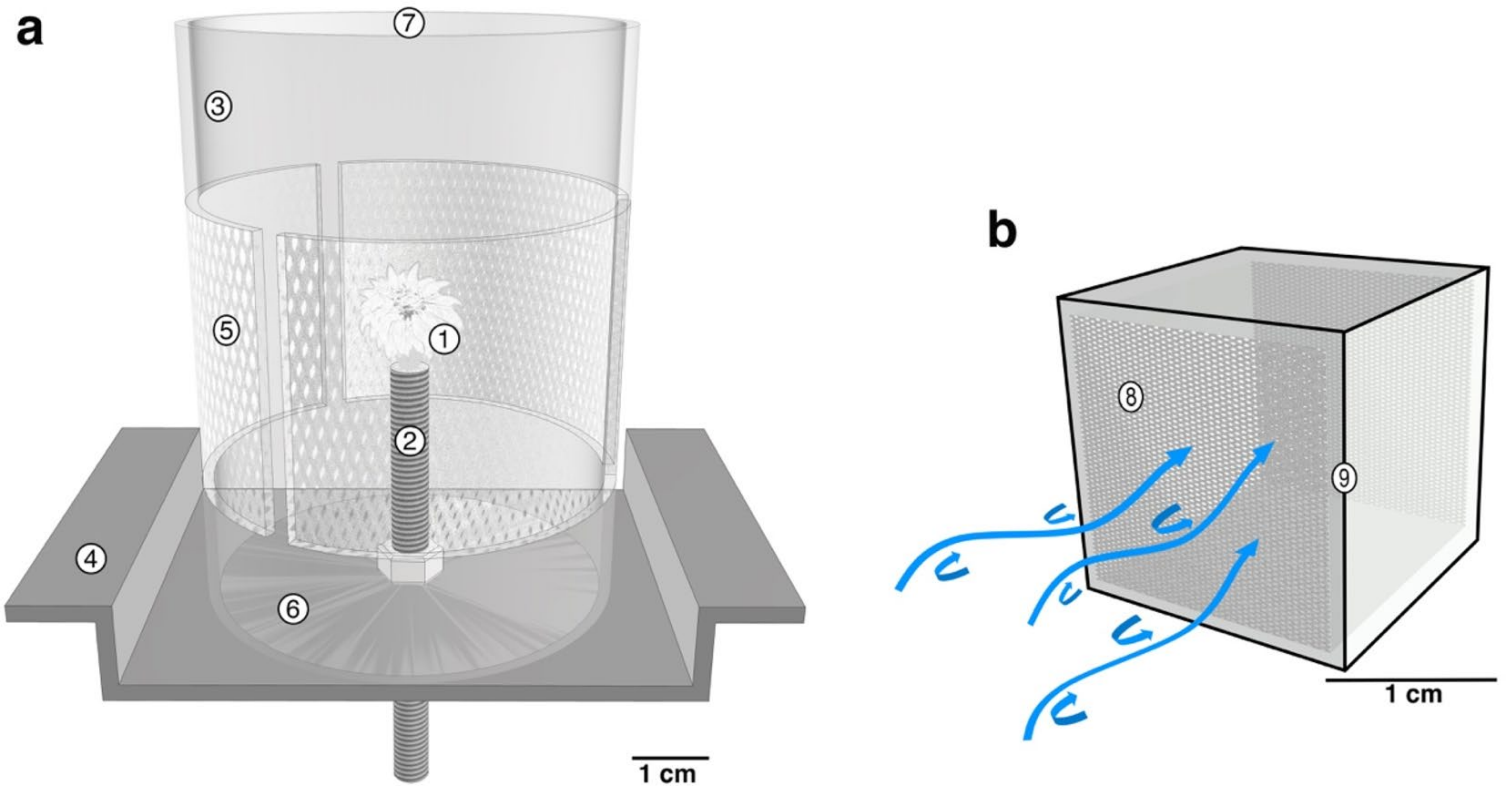
Schematic drawing of the (**a**) cylindrical plastic container and the (**b**) flow-through cube in the holding tank. (**a**) Reproductive polyp (1) glued onto a polyethylene screw (2), and placed in a cylindrical (7.2 cm high, 5.4 cm ø) plastic container (3), which was screwed onto a plastic holder (4). The plastic container had three side windows covered with mesh (100 µm) (5) to allow water exchange. A closed bottom (6) and an open top (7), which rose 1 cm above the water surface ensured retention of larvae. (**b**) Larvae collected from the container were placed into a cube with the upstream and downstream side covered with a 100 µm mesh (8), whereas the top, floor and walls consisted of coverslips glued with silicone (9, black line). Blue arrows indicate the direction of the water flow with eddies and vortices.

After arrival, the live corals were glued to plastic screws (Fig. 8a) using Preis Easy Glue Underwater (Preis Aquaristik KG, Bayerfeld, Germany), and placed in a 285 L closed-circuit holding tank with artificial seawater (Dupla Marin Premium Reef Salt, Dohse Aquaristik, Grafschaft-Gelsdorf, Germany), which had similar characteristics to those at the sampling site and depth, analogous to the study of Laudien et al.^59^ (see their Suppl. M. 1). The aquarium was connected to a large tank (80 L) with a water flow of 5–10 L min^-1^, in which an aquarium pump (Turbelle® stream 6105, Tunze Aquarientechnik Gmbh, Penzberg, Germany) ensured a counterclockwise current of 2.15 ± 1.03 cm s^-1^ with discrete eddies and vortices. The salinity of the UV-light treated seawater of 31.7 ± 0.6 was maintained at 11.5 ± 0.8 °C with a pH of 7.9 ± 0.1 (WTW conductivity meter, ProfiLab24 GmbH, Germany), and an oxygen concentration of 100% saturation. The total alkalinity (TA) of the seawater was analysed once a week and measured according to Dickson et al.^75^. Aragonite saturation state (Ω_ar_) was maintained at 1.7 ± 0.4 and calculated from pH, TA, salinity, and temperature using the program CO2SYS^76^. Immediately before the weekly water exchange, nutrient concentrations (nitrate, nitrite, phosphate, and ammonium) were determined using a photometric test (Spectroquant® test, Merck KGaA, Darmstadt, Germany).

The diet consisted of live *Artemia franciscana*-nauplii and *A. persimilis*-nauplii (ca. 1200 ind. L^−1^) three times a week and one thawed juvenile krill (*Euphausia pacifica,* Zierfischfutterhandel Norbert Erdmann e.K., Ritterhude, Germany) per polyp once a week throughout the study period. As *C. huinayensis* populations also occur in the aphotic zone^36^, it could be inferred that a light-dark cycle is not a direct environmental factor in the reproductive cycle of this species. Hence, corals were kept in the dark to avoid fouling by cyanobacteria and macroalgae on the aquarium walls, thus decreasing coral stress by reducing regular cleanings.

### Collection, characterization, and settlement of larvae

To investigate the reproductive mode and larval released periodicity, seven adult polyps used throughout the study were placed in individual plastic cylinders fixed on horizontal plastic holders, and immersed in the holding tank to allow retention and collection of larvae (Fig. 8a). Thanks to the translucent tissue, planulae were observed in the tentacles and gastrovascular area, which were collected at the time of release into the cylinders with a plastic pipette (3 ml) and counted twice a day from Monday to Friday to calculate the production rate of each individual polyp. A total of 27 flow-through glass cubes of 2 × 2 × 2 cm (Fig. 8b) were utilized for larval characterization and for the description of metamorphosis. The cubes consisted of four coverslips oriented parallel to the water flow and two 100 µm mesh screens intercepting the flow at the upstream and downstream face of the cube. The coverslips were glued with silicon (Silexo, Juwel Aquarium AG & Co. KG, Germany). To describe the larval behaviour, i.e., planktonic stage, 3–8 larvae were placed in two cubes and kept there until all larvae attached to the substrate. To determine the possible influence of natural biofilm on the substrate on larval settlement, seven cubes were left in the holding tank for at least 15 days before introducing 3–8 larvae, and four cubes were inserted into the tank at the same time as 3–8 larvae were introduced into them. Swimming velocities were calculated from video tracking of 12 larva inside the cubes. Video clips (3–10 s) recorded with a Nikon D7000 DSLR and a 60 mm lens at 24 fps were played frame by frame (spatial resolution of 1920 × 1080 px) and the displacement of the center of the planula between frames was recorded to the nearest 0.1 mm. A scale bar was placed next to the cube for calibration. Furthermore, a total of 12 larvae were photographed to determine their length and width using Adobe Photoshop® CC (San Jose, California, USA).

Fourteen cubes with 3–8 larvae inside were used to describe the metamorphosis and growth of juveniles. Larval attachment was considered successful if the diameter of a larva doubled in one day. Thereafter, the cube was disassembled and the settled recruit was placed in a respective glass petri dish (6 cm ø) to allow handling of the recruit without air exposure. Unsettled larvae from the cubes were released into the holding tank.

Metamorphosis and post-larval development stages were documented by inverting the coverslip in the glass Petri dish and taking photos at the oral and aboral poles of the polyps. To prevent crushing of the inverted polyp, a smaller Petri dish (0.5 cm height, 2 cm ø) holding the coverslip with the polyp facing downward was used as a spacer. Photos were taken daily for the first two weeks, then once a week for three years. The photos were taken from the same distance and angle using a Leica camera (IC80 HD; Wetzlar, Germany) connected to a stereomicroscope (Leica MZ 16, Wetzlar, Germany).

### Histology

The 60 corals preserved in Formalin were decalcified in Rapid Bone Decalcifying solution (Thermo Scientific™ Shandon™ TBD-1™, Tudor Road, UK) for 30 min to dissolve the skeleton. For dehydration, the skeleton-free tissue was placed in 70–100% ethanol, with 10% increments every 1 h. Tissue was then immersed in Xylene twice for 1 h. The tissue was then transferred to paraffin wax (Polarit, Labortechnik Süsse, Hessen, German) in an oven at 60 °C, where it underwent four wax changes over a period of 2.6 h. After cooling, the wax blocks were cut into 3 μm thick sections, leaving a distance of 45 μm between sections, based on the mean width of the *C. huinayensis* oocyte nucleolus. The sections were then mounted on glass slides and stained with haematoxylin-eosin. Histological sections were examined under a Zeiss Axioscope microscope with an Olympus (DP70) camera mounted.

## Data Availability

The larval released dataset and raw images of Figs. 5 and 6 generated during the current study are available in the data repository www.PANGAEA.de at 10.1594/PANGAEA.948437, 10.1594/PANGAEA.948397, 10.1594/PANGAEA.948434, respectively.
